# Crystal structure of *N*-[(naphthalen-1-yl)carbamo­thio­yl]cyclo­hexa­necarboxamide

**DOI:** 10.1107/S2056989015011950

**Published:** 2015-06-27

**Authors:** G. Vimala, J. Haribabu, S. Aishwarya, R. Karvembu, A. SubbiahPandi

**Affiliations:** a Department of Physics, Presidency College (Autonomous), Chennai 600 005, India; b Department of Chemistry, National Institute of Technology, Trichy 620 015, India

**Keywords:** crystal structure, whole-mol­ecule disorder, thio­urea derivatives, intra­molecular N—H⋯O hydrogen bond, N—H⋯S hydrogen bonds, π–π stacking inter­actions, C—H⋯π inter­actions

## Abstract

The title compound, C_18_H_20_N_2_OS, displays whole-mol­ecule disorder over two adjacent sets of sites with an occupancy ratio of 0.630 (11):0.370 (11). In each disorder component, the cyclo­hexyl ring shows a chair conformation with the exocyclic C—C bond in an equatorial orientation. The dihedral angles between the cyclo­hexyl ring (all atoms) and the naphthyl ring system are 36.9 (6) for the major component and 20.7 (12)° for the minor component. Each component features an intra­molecular N—H⋯O hydrogen bond, which closes an *S*(5) ring. In the crystal, inversion dimers linked by pairs of N—H⋯S hydrogen bonds generate *R* ^2^_2_(8) loops for both components. Aromatic π–π stacking inter­actions [shortest centroid–centroid separation = 3.593 (9) Å] and a C—H⋯π inter­action are also observed.

## Related literature   

For background to the varied properties of thio­urea derivatives, see: Sun *et al.* (2006 ▸); Shen *et al.* (2006 ▸). For related structures, see: Hu *et al.* (2011 ▸); Gangadharan *et al.* (2015 ▸).
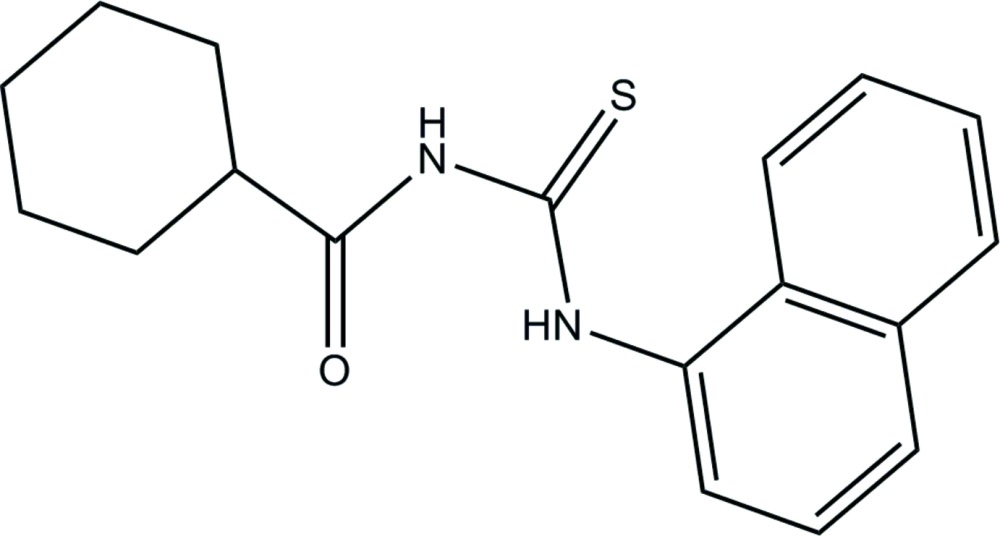


## Experimental   

### Crystal data  

C_18_H_20_N_2_OS
*M* *_r_* = 312.42
Triclinic, 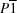

*a* = 7.0464 (5) Å
*b* = 11.0379 (5) Å
*c* = 12.4151 (8) Å
α = 110.873 (3)°
β = 100.660 (3)°
γ = 104.022 (3)°
*V* = 835.24 (9) Å^3^
*Z* = 2
Mo *K*α radiation
μ = 0.20 mm^−1^
*T* = 293 K
0.35 × 0.30 × 0.25 mm

### Data collection  

Bruker APEXII CCD diffractometer
Absorption correction: multi-scan (*SADABS*; Bruker, 2004 ▸) *T*_min_ = 0.934, *T*_max_ = 0.952
14064 measured reflections
2945 independent reflections
1930 reflections with *I* > 2σ(*I*)
*R*_int_ = 0.031

### Refinement  

*R*[*F* ^2^ > 2σ(*F* ^2^)] = 0.068
*wR*(*F* ^2^) = 0.219
*S* = 1.05
2945 reflections
404 parameters
1056 restraints
H atoms treated by a mixture of independent and constrained refinement
Δρ_max_ = 0.87 e Å^−3^
Δρ_min_ = −0.25 e Å^−3^

### 

Data collection: *APEX2* (Bruker, 2004 ▸); cell refinement: *SAINT* (Bruker, 2004 ▸); data reduction: *SAINT*; program(s) used to solve structure: *SHELXS97* (Sheldrick, 2008 ▸); program(s) used to refine structure: *SHELXL97* (Sheldrick, 2008 ▸); molecular graphics: *ORTEP-3 for Windows* (Farrugia, 2012 ▸); software used to prepare material for publication: *PLATON* (Spek, 2009 ▸).

## Supplementary Material

Crystal structure: contains datablock(s) global, I. DOI: 10.1107/S2056989015011950/hb7448sup1.cif

Structure factors: contains datablock(s) I. DOI: 10.1107/S2056989015011950/hb7448Isup2.hkl

Supporting information file. DOI: 10.1107/S2056989015011950/hb7448Isup3.cml

**Fig. 1.**
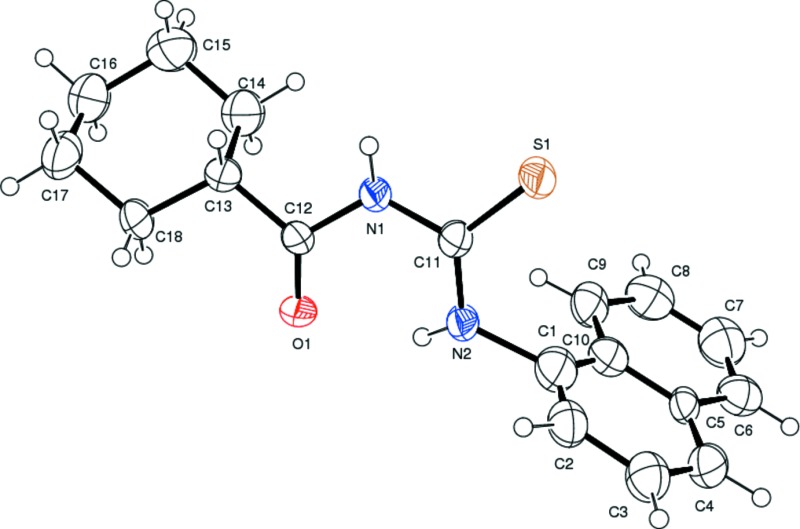
The molecular structure of the major component of the title compound, with displacement ellipsoids drawn at 40% probability level.

. DOI: 10.1107/S2056989015011950/hb7448fig1.tif
The mol­ecular structure of the major component of the title compound, with displacement ellipsoids drawn at 40% probability level.

**Fig. 2.**
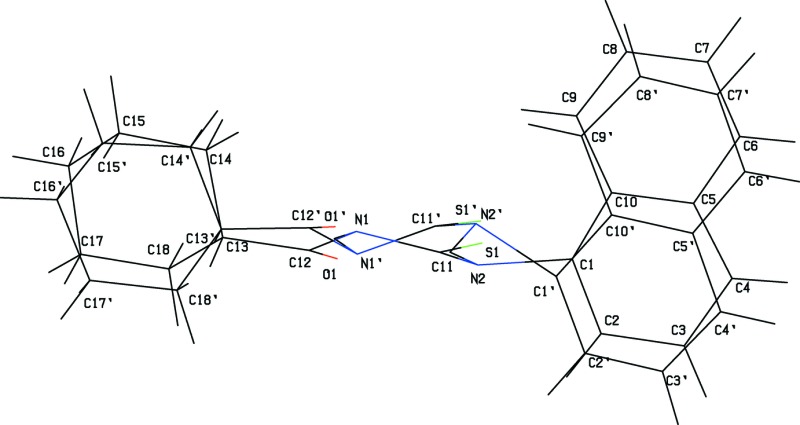
Stick plot of both major and minor components of the title compound, with the atoms label for non-H atoms.

**Fig. 3.**
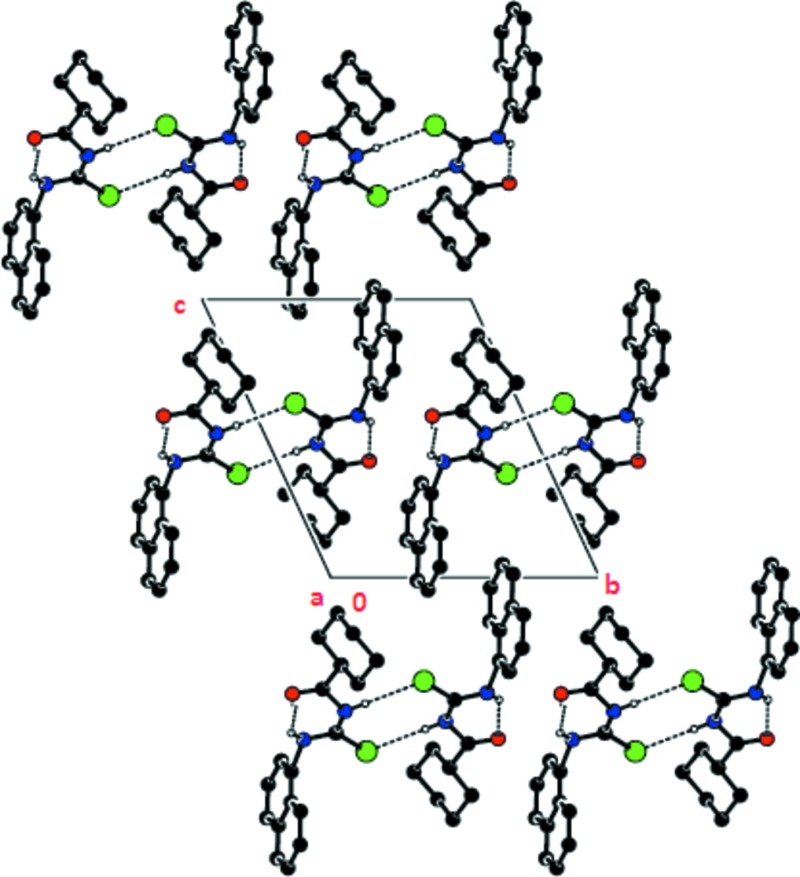
The crystal packing of the title compound, viewed along the b axis. The hydrogen bonds are shown as dashed lines (see Table 1 for details).

. DOI: 10.1107/S2056989015011950/hb7448fig2.tif
Stick plot of both major and minor components of the title compound, with the atoms label for non-H atoms.

b . DOI: 10.1107/S2056989015011950/hb7448fig3.tif
The crystal packing of the title compound, viewed along the *b* axis. The hydrogen bonds are shown as dashed lines (see Table 1 for details).

CCDC reference: 1408027

Additional supporting information:  crystallographic information; 3D view; checkCIF report

## Figures and Tables

**Table 1:** Hydrogen-bond geometry (, ) *Cg*1 is the centroid of the C1C5/C10 ring.

*D*H*A*	*D*H	H*A*	*D* *A*	*D*H*A*
N2H2*A*O1	0.88(4)	1.97(4)	2.667(15)	135(4)
N2H2*A*O1	0.93(4)	2.03(4)	2.62(3)	120(4)
N1H1*A*S1^i^	0.87(3)	2.53(3)	3.370(19)	161(4)
N1H1*A*S1^i^	0.90(4)	2.59(4)	3.44(3)	159(4)
C18H18*B* *Cg*1^ii^	0.90	2.66	3.527(2)	148

Symmetry codes: (i) 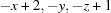; (ii) 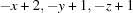.

## supplementary crystallographic information

### S1. Comment

The design and synthesis of thiourea are of considerable interest because of their use in agriculture, medicine and analytical chemistry (J·H. Hu *et al.*, 2011). Thiourea derivatives are driven by their potential as biological active compounds (Sun *et al.*, 2006) and in the material application such as anti corrosion (Shen *et al.*, 2006). As part of our own studies in this area, the crystal structure of the title compound has been determined and the results are presented herein.

For the major disorder component, the cyclohexane ring (C13—C18) adopts chair conformation [puckering amplitudes and smallest displacement parameters are q = 0.568 Å, θ = 177.7 (8)°, φ = 19 (29)° and ΔC_s_ = 0.9 (14) Å]. Similarly, for the minor disorder component, the cyclohexane ring (C13^'^—C18^'^) adopts a chair conformation. [puckering amplitudes and smallest displacement parameters are q = 0.56 (3) Å, θ = 180 (3)°, φ = 354 (31)° and ΔC_s_ = 3.0 (4) Å]. The dihedral angles between cyclohexane and benzene rings (C5—C8/C10) and (C5—C10) of naphthalene moiety are 37.0 (7)and 36.5 (7)° (major component). In the case of minor component, the dihedral angles between cyclohexane and benzene rings (C5—C8/C10)and (C5—C10) of naphthalene are 37.0 (7)and 36.5 (7)°, respectively. The molecular conformation is consolidated by an intramolecular N—H···O hydrogen bond, forming S(5) ring motif.

The crystal packing features N—H···S hydrogen bonds with the symmetry code: (i) 2 - *x*,-*y*,1 - *z*, which links the molecules into centrosymmetric dimers with graph-set descriptor of *R*^2^_2_(8). The crystal packing also features C—H···π (Table 1) and π-π interactions (*Cg*2···*Cg*2^ii^ = 3.593 (9) Å; *Cg*2 is the centroid of a ring C5—C10; symmetry code: (ii) 3 - *x*,1 - *y*,2 - *z*. The packing view of the title compound is shown in Fig. 3.

### S2. Experimental

A solution of cyclohexane carbonyl chloride (1.4661 g, 10 mmol) in acetone (60 ml) was added drop wise to a suspension of potassium thiocyanate (0.9718 g, 10 mmol) in anhydrous acetone (60 ml). The reaction mixture was heated under reflux for 45 minutes and then cooled to room temperature. A solution of substituted naphthalen-1-amine (1.43 g, 10 mmol) in acetone (60 ml) was added and the resulting mixture was stirred for 2 h at room temperature. Hydrochloric acid (0.1 N, 500 ml) was added and the resulting white solid was filtered off, washed with water and dried in vaccum. The yield of the isolated product was 89%, giving colourless blocks.

### S3. Refinement

All H atoms were fixed geometrically and allowed to ride on their parent C atoms, with C—H distances fixed in the range 0.93–0.97 Å with *U*_iso_(H) = 1.5*U*_eq_(C) for methyl H atoms and 1.2*U*_eq_(C) for all other H atoms.

### Crystal data

**Table:**

C_18_H_20_N_2_OS	*Z* = 2
*M**_r_* = 312.42	*F*(000) = 332
Triclinic, *P*1	*D*_x_ = 1.242 Mg m^−^^3^
Hall symbol: -P 1	Mo *K*α radiation, λ = 0.71073 Å
*a* = 7.0464 (5) Å	Cell parameters from 1930 reflections
*b* = 11.0379 (5) Å	θ = 2.1–25.0°
*c* = 12.4151 (8) Å	µ = 0.20 mm^−^^1^
α = 110.873 (3)°	*T* = 293 K
β = 100.660 (3)°	Block, white
γ = 104.022 (3)°	0.35 × 0.30 × 0.25 mm
*V* = 835.24 (9) Å^3^	

### Data collection

**Table:**

Bruker APEXII CCD diffractometer	2945 independent reflections
Radiation source: fine-focus sealed tube	1930 reflections with *I* > 2σ(*I*)
Graphite monochromator	*R*_int_ = 0.031
ω and φ scan	θ_max_ = 25.0°, θ_min_ = 2.1°
Absorption correction: multi-scan (*SADABS*; Bruker, 2004)	*h* = −8→8
*T*_min_ = 0.934, *T*_max_ = 0.952	*k* = −13→13
14064 measured reflections	*l* = −14→14

### Refinement

**Table:**

Refinement on *F*^2^	Primary atom site location: structure-invariant direct methods
Least-squares matrix: full	Secondary atom site location: difference Fourier map
*R*[*F*^2^ > 2σ(*F*^2^)] = 0.068	Hydrogen site location: inferred from neighbouring sites
*wR*(*F*^2^) = 0.219	H atoms treated by a mixture of independent and constrained refinement
*S* = 1.05	*w* = 1/[σ^2^(*F*_o_^2^) + (0.0972*P*)^2^ + 1.1049*P*] where *P* = (*F*_o_^2^ + 2*F*_c_^2^)/3
2945 reflections	(Δ/σ)_max_ = 0.053
404 parameters	Δρ_max_ = 0.87 e Å^−^^3^
1056 restraints	Δρ_min_ = −0.25 e Å^−^^3^

### Special details

**Table:**

Geometry. All e.s.d.'s (except the e.s.d. in the dihedral angle between two l.s. planes) are estimated using the full covariance matrix. The cell e.s.d.'s are taken into account individually in the estimation of e.s.d.'s in distances, angles and torsion angles; correlations between e.s.d.'s in cell parameters are only used when they are defined by crystal symmetry. An approximate (isotropic) treatment of cell e.s.d.'s is used for estimating e.s.d.'s involving l.s. planes.
Refinement. Refinement of *F*^2^ against ALL reflections. The weighted *R*-factor *wR* and goodness of fit *S* are based on *F*^2^, conventional *R*-factors *R* are based on *F*, with *F* set to zero for negative *F*^2^. The threshold expression of *F*^2^ > σ(*F*^2^) is used only for calculating *R*-factors(gt) *etc*. and is not relevant to the choice of reflections for refinement. *R*-factors based on *F*^2^ are statistically about twice as large as those based on *F*, and *R*- factors based on ALL data will be even larger.

### Fractional atomic coordinates and isotropic or equivalent isotropic displacement parameters (Å^2^)

**Table:**

	*x*	*y*	*z*	*U*_iso_*/*U*_eq_	Occ. (<1)
C1	1.4139 (12)	0.4829 (15)	0.6995 (10)	0.072 (3)	0.630 (11)
C2	1.5803 (14)	0.5008 (15)	0.6610 (8)	0.058 (2)	0.630 (11)
H2	1.5690	0.4550	0.5798	0.069*	0.630 (11)
C3	1.7732 (13)	0.5902 (11)	0.7454 (8)	0.064 (2)	0.630 (11)
H3	1.8908	0.6065	0.7214	0.076*	0.630 (11)
C4	1.7793 (14)	0.6525 (12)	0.8658 (8)	0.065 (2)	0.630 (11)
H4	1.9016	0.7111	0.9257	0.078*	0.630 (11)
C5	1.5918 (13)	0.6230 (12)	0.8936 (7)	0.0446 (17)	0.630 (11)
C6	1.5952 (14)	0.6835 (11)	1.0116 (8)	0.062 (2)	0.630 (11)
H6	1.7193	0.7418	1.0700	0.075*	0.630 (11)
C7	1.4217 (16)	0.6606 (16)	1.0460 (8)	0.082 (3)	0.630 (11)
H7	1.4294	0.7032	1.1270	0.099*	0.630 (11)
C8	1.2357 (16)	0.5753 (16)	0.9622 (9)	0.076 (3)	0.630 (11)
H8	1.1199	0.5574	0.9875	0.091*	0.630 (11)
C9	1.2189 (15)	0.5164 (15)	0.8419 (8)	0.062 (2)	0.630 (11)
H9	1.0919	0.4657	0.7838	0.074*	0.630 (11)
C10	1.4035 (19)	0.536 (2)	0.8090 (10)	0.063 (3)	0.630 (11)
C11	1.1480 (17)	0.2490 (13)	0.5594 (10)	0.042 (2)	0.630 (11)
C12	0.839 (2)	0.2236 (13)	0.4017 (11)	0.042 (3)	0.630 (11)
C13	0.643 (2)	0.113 (2)	0.3153 (11)	0.052 (3)	0.630 (11)
H13	0.6743	0.0287	0.2776	0.062*	0.630 (11)
C14	0.4915 (15)	0.0832 (11)	0.3824 (10)	0.067 (2)	0.630 (11)
H14A	0.4669	0.1674	0.4258	0.080*	0.630 (11)
H14B	0.5514	0.0538	0.4413	0.080*	0.630 (11)
C15	0.2882 (18)	−0.0269 (11)	0.2994 (11)	0.084 (3)	0.630 (11)
H15A	0.1929	−0.0367	0.3455	0.101*	0.630 (11)
H15B	0.3083	−0.1146	0.2629	0.101*	0.630 (11)
C16	0.2014 (19)	0.0145 (14)	0.2016 (11)	0.088 (3)	0.630 (11)
H16A	0.0733	−0.0568	0.1475	0.106*	0.630 (11)
H16B	0.1720	0.0986	0.2389	0.106*	0.630 (11)
C17	0.3457 (16)	0.0371 (10)	0.1289 (11)	0.074 (3)	0.630 (11)
H17A	0.2864	0.0664	0.0697	0.088*	0.630 (11)
H17B	0.3691	−0.0479	0.0865	0.088*	0.630 (11)
C18	0.5478 (15)	0.1477 (9)	0.2156 (9)	0.059 (3)	0.630 (11)
H18A	0.6436	0.1599	0.1703	0.071*	0.630 (11)
H18B	0.5240	0.2342	0.2514	0.071*	0.630 (11)
S1	1.2576 (18)	0.1673 (11)	0.6264 (10)	0.0567 (16)	0.630 (11)
N1	0.938 (2)	0.1781 (12)	0.4817 (12)	0.044 (3)	0.630 (11)
N2	1.2224 (9)	0.3819 (7)	0.5845 (5)	0.0416 (16)	0.630 (11)
O1	0.886 (2)	0.3438 (12)	0.4191 (12)	0.055 (3)	0.630 (11)
C1'	1.4044 (16)	0.4630 (19)	0.6642 (11)	0.032 (3)	0.370 (11)
C2'	1.574 (2)	0.480 (3)	0.6233 (13)	0.063 (4)	0.370 (11)
H2'	1.5553	0.4330	0.5412	0.076*	0.370 (11)
C3'	1.7640 (19)	0.5594 (19)	0.6957 (14)	0.060 (4)	0.370 (11)
H3'	1.8726	0.5649	0.6630	0.072*	0.370 (11)
C4'	1.802 (2)	0.635 (2)	0.8205 (14)	0.070 (4)	0.370 (11)
H4'	1.9328	0.6931	0.8711	0.084*	0.370 (11)
C5'	1.632 (3)	0.618 (3)	0.8638 (14)	0.067 (4)	0.370 (11)
C6'	1.656 (3)	0.682 (3)	0.9838 (16)	0.097 (5)	0.370 (11)
H6'	1.7861	0.7371	1.0373	0.117*	0.370 (11)
C7'	1.489 (3)	0.666 (3)	1.0264 (14)	0.077 (5)	0.370 (11)
H7'	1.5100	0.7078	1.1092	0.092*	0.370 (11)
C8'	1.298 (3)	0.593 (3)	0.9538 (14)	0.070 (4)	0.370 (11)
H8'	1.1874	0.5978	0.9844	0.084*	0.370 (11)
C9'	1.261 (3)	0.507 (3)	0.8293 (12)	0.076 (5)	0.370 (11)
H9'	1.1367	0.4381	0.7805	0.091*	0.370 (11)
C10'	1.434 (2)	0.536 (3)	0.7873 (12)	0.037 (3)	0.370 (11)
C11'	1.100 (3)	0.253 (2)	0.5789 (19)	0.049 (4)	0.370 (11)
C12'	0.809 (3)	0.221 (2)	0.424 (2)	0.042 (4)	0.370 (11)
C13'	0.629 (3)	0.110 (4)	0.3220 (19)	0.051 (4)	0.370 (11)
H13'	0.6531	0.0227	0.3069	0.061*	0.370 (11)
C14'	0.444 (3)	0.103 (3)	0.364 (2)	0.096 (5)	0.370 (11)
H14C	0.4248	0.1923	0.3884	0.116*	0.370 (11)
H14D	0.4647	0.0828	0.4344	0.116*	0.370 (11)
C15'	0.256 (4)	−0.005 (3)	0.2673 (18)	0.102 (6)	0.370 (11)
H15C	0.2702	−0.0944	0.2488	0.123*	0.370 (11)
H15D	0.1378	−0.0039	0.2970	0.123*	0.370 (11)
C16'	0.219 (3)	0.017 (2)	0.1542 (17)	0.081 (5)	0.370 (11)
H16C	0.1000	−0.0564	0.0919	0.098*	0.370 (11)
H16D	0.1951	0.1042	0.1694	0.098*	0.370 (11)
C17'	0.408 (3)	0.018 (2)	0.116 (2)	0.090 (6)	0.370 (11)
H17C	0.3886	0.0311	0.0420	0.108*	0.370 (11)
H17D	0.4230	−0.0711	0.0981	0.108*	0.370 (11)
C18'	0.602 (3)	0.126 (2)	0.2064 (17)	0.083 (5)	0.370 (11)
H18C	0.7176	0.1164	0.1762	0.099*	0.370 (11)
H18D	0.5957	0.2172	0.2197	0.099*	0.370 (11)
S1'	1.222 (3)	0.1767 (19)	0.6457 (17)	0.065 (4)	0.370 (11)
N2'	1.1590 (19)	0.3881 (14)	0.6219 (11)	0.083 (4)	0.370 (11)
N1'	0.972 (3)	0.176 (2)	0.461 (2)	0.036 (4)	0.370 (11)
O1'	0.838 (4)	0.341 (2)	0.448 (2)	0.057 (4)	0.370 (11)
H1A	0.918 (6)	0.0899 (17)	0.454 (4)	0.069*	
H2A	1.129 (5)	0.415 (4)	0.560 (3)	0.069*	

### Atomic displacement parameters (Å^2^)

**Table:**

	*U*^11^	*U*^22^	*U*^33^	*U*^12^	*U*^13^	*U*^23^
C1	0.079 (5)	0.052 (5)	0.075 (7)	0.020 (4)	−0.001 (5)	0.030 (6)
C2	0.071 (4)	0.050 (5)	0.046 (5)	0.024 (3)	0.007 (4)	0.017 (4)
C3	0.072 (4)	0.066 (6)	0.051 (6)	0.026 (4)	0.016 (4)	0.021 (5)
C4	0.054 (4)	0.058 (5)	0.075 (5)	0.014 (3)	−0.001 (4)	0.034 (5)
C5	0.052 (4)	0.031 (3)	0.039 (4)	0.011 (3)	−0.008 (3)	0.013 (3)
C6	0.070 (5)	0.057 (4)	0.070 (4)	0.028 (5)	0.022 (4)	0.033 (4)
C7	0.104 (7)	0.077 (5)	0.068 (5)	0.042 (6)	0.022 (5)	0.027 (4)
C8	0.084 (6)	0.081 (6)	0.080 (5)	0.037 (5)	0.036 (4)	0.043 (4)
C9	0.064 (5)	0.056 (4)	0.066 (4)	0.035 (4)	0.004 (3)	0.024 (3)
C10	0.074 (5)	0.051 (4)	0.063 (5)	0.017 (4)	0.005 (4)	0.032 (4)
C11	0.047 (5)	0.037 (3)	0.035 (4)	0.011 (3)	0.006 (3)	0.012 (3)
C12	0.053 (5)	0.037 (3)	0.032 (4)	0.008 (3)	0.007 (3)	0.017 (3)
C13	0.058 (4)	0.036 (4)	0.053 (4)	0.009 (4)	−0.001 (4)	0.022 (3)
C14	0.058 (5)	0.069 (4)	0.070 (5)	0.021 (3)	0.011 (4)	0.031 (3)
C15	0.066 (5)	0.082 (5)	0.099 (6)	0.007 (4)	0.013 (5)	0.049 (4)
C16	0.063 (5)	0.092 (5)	0.089 (7)	0.005 (4)	−0.012 (5)	0.046 (5)
C17	0.072 (6)	0.056 (4)	0.070 (5)	0.018 (4)	−0.013 (5)	0.020 (4)
C18	0.066 (5)	0.050 (4)	0.055 (4)	0.016 (3)	−0.009 (3)	0.031 (3)
S1	0.069 (3)	0.035 (2)	0.054 (3)	0.0193 (14)	−0.0060 (17)	0.017 (2)
N1	0.056 (5)	0.031 (3)	0.036 (5)	0.009 (3)	−0.001 (4)	0.013 (3)
N2	0.047 (3)	0.033 (3)	0.038 (3)	0.013 (2)	0.001 (2)	0.014 (2)
O1	0.060 (6)	0.036 (3)	0.059 (6)	0.002 (3)	−0.004 (3)	0.028 (3)
C1'	0.049 (5)	0.025 (5)	0.015 (4)	0.012 (4)	−0.004 (3)	0.007 (4)
C2'	0.085 (6)	0.051 (7)	0.041 (7)	0.021 (5)	0.001 (5)	0.014 (6)
C3'	0.053 (6)	0.057 (7)	0.063 (8)	0.018 (5)	0.010 (6)	0.020 (7)
C4'	0.080 (7)	0.060 (7)	0.057 (9)	0.026 (6)	0.004 (7)	0.018 (8)
C5'	0.076 (7)	0.051 (5)	0.060 (7)	0.019 (6)	0.001 (6)	0.020 (6)
C6'	0.103 (9)	0.075 (7)	0.090 (8)	0.025 (8)	−0.001 (7)	0.027 (7)
C7'	0.084 (10)	0.075 (7)	0.080 (7)	0.032 (9)	0.027 (7)	0.038 (6)
C8'	0.079 (8)	0.074 (7)	0.063 (6)	0.028 (7)	0.030 (6)	0.030 (5)
C9'	0.058 (7)	0.055 (6)	0.112 (7)	0.018 (7)	0.004 (6)	0.044 (6)
C10'	0.049 (5)	0.022 (4)	0.025 (5)	0.007 (4)	−0.011 (4)	0.006 (5)
C11'	0.056 (7)	0.032 (5)	0.050 (7)	0.023 (5)	−0.002 (6)	0.011 (5)
C12'	0.053 (6)	0.033 (5)	0.040 (7)	0.008 (5)	0.007 (5)	0.022 (5)
C13'	0.053 (6)	0.041 (6)	0.052 (6)	0.010 (6)	−0.003 (6)	0.024 (5)
C14'	0.073 (8)	0.101 (8)	0.084 (7)	0.009 (7)	0.012 (6)	0.024 (7)
C15'	0.079 (8)	0.104 (8)	0.100 (9)	−0.005 (7)	0.013 (7)	0.047 (7)
C16'	0.069 (7)	0.072 (6)	0.082 (9)	0.011 (6)	−0.005 (8)	0.031 (7)
C17'	0.074 (8)	0.082 (8)	0.074 (7)	0.019 (7)	−0.011 (7)	0.008 (6)
C18'	0.064 (8)	0.083 (8)	0.066 (7)	0.014 (6)	−0.001 (6)	0.010 (6)
S1'	0.074 (7)	0.033 (2)	0.060 (6)	0.020 (3)	−0.016 (4)	0.006 (3)
N2'	0.097 (7)	0.040 (5)	0.065 (7)	0.020 (6)	−0.041 (5)	0.006 (5)
N1'	0.045 (6)	0.028 (4)	0.031 (6)	0.011 (4)	0.009 (4)	0.009 (4)
O1'	0.061 (10)	0.041 (5)	0.058 (9)	0.007 (6)	−0.003 (6)	0.025 (5)

### Geometric parameters (Å, º)

**Table:**

C1—C10	1.298 (10)	C1'—C2'	1.378 (11)
C1—C2	1.344 (9)	C1'—C10'	1.396 (12)
C1—N2	1.589 (8)	C1'—N2'	1.616 (10)
C2—C3	1.416 (9)	C2'—C3'	1.341 (13)
C2—H2	0.9300	C2'—H2'	0.9300
C3—C4	1.392 (8)	C3'—C4'	1.408 (11)
C3—H3	0.9300	C3'—H3'	0.9300
C4—C5	1.418 (9)	C4'—C5'	1.399 (14)
C4—H4	0.9300	C4'—H4'	0.9300
C5—C6	1.370 (9)	C5'—C6'	1.359 (14)
C5—C10	1.392 (11)	C5'—C10'	1.399 (15)
C6—C7	1.364 (9)	C6'—C7'	1.378 (12)
C6—H6	0.9300	C6'—H6'	0.9300
C7—C8	1.373 (10)	C7'—C8'	1.336 (14)
C7—H7	0.9300	C7'—H7'	0.9300
C8—C9	1.369 (9)	C8'—C9'	1.432 (13)
C8—H8	0.9300	C8'—H8'	0.9300
C9—C10	1.424 (10)	C9'—C10'	1.422 (14)
C9—H9	0.9300	C9'—H9'	0.9300
C11—N2	1.329 (14)	C11'—N2'	1.31 (2)
C11—N1	1.451 (19)	C11'—N1'	1.40 (3)
C11—S1	1.653 (6)	C11'—S1'	1.652 (8)
C12—O1	1.214 (6)	C12'—O1'	1.214 (8)
C12—N1	1.411 (9)	C12'—N1'	1.413 (11)
C12—C13	1.502 (7)	C12'—C13'	1.500 (9)
C13—C18	1.511 (10)	C13'—C14'	1.490 (14)
C13—C14	1.512 (8)	C13'—C18'	1.491 (13)
C13—H13	0.9800	C13'—H13'	0.9800
C14—C15	1.520 (8)	C14'—C15'	1.502 (13)
C14—H14A	0.9700	C14'—H14C	0.9700
C14—H14B	0.9700	C14'—H14D	0.9700
C15—C16	1.521 (8)	C15'—C16'	1.495 (13)
C15—H15A	0.9700	C15'—H15C	0.9700
C15—H15B	0.9700	C15'—H15D	0.9700
C16—C17	1.512 (9)	C16'—C17'	1.492 (14)
C16—H16A	0.9700	C16'—H16C	0.9700
C16—H16B	0.9700	C16'—H16D	0.9700
C17—C18	1.526 (8)	C17'—C18'	1.498 (13)
C17—H17A	0.9700	C17'—H17C	0.9700
C17—H17B	0.9700	C17'—H17D	0.9700
C18—H18A	0.9700	C18'—H18C	0.9700
C18—H18B	0.9700	C18'—H18D	0.9700
N1—H1A	0.874 (19)	N2'—H2A	0.927 (18)
N2—H2A	0.883 (18)	N1'—H1A	0.906 (19)
			
C10—C1—C2	128.3 (8)	C2'—C1'—C10'	117.6 (9)
C10—C1—N2	124.4 (8)	C2'—C1'—N2'	143.7 (10)
C2—C1—N2	107.4 (7)	C10'—C1'—N2'	98.7 (8)
C1—C2—C3	119.2 (6)	C3'—C2'—C1'	123.4 (8)
C1—C2—H2	120.4	C3'—C2'—H2'	118.3
C3—C2—H2	120.4	C1'—C2'—H2'	118.3
C4—C3—C2	117.2 (6)	C2'—C3'—C4'	121.2 (8)
C4—C3—H3	121.4	C2'—C3'—H3'	119.4
C2—C3—H3	121.4	C4'—C3'—H3'	119.4
C3—C4—C5	117.5 (6)	C5'—C4'—C3'	116.1 (10)
C3—C4—H4	121.3	C5'—C4'—H4'	122.0
C5—C4—H4	121.3	C3'—C4'—H4'	121.9
C6—C5—C10	117.4 (6)	C6'—C5'—C10'	117.6 (11)
C6—C5—C4	118.2 (6)	C6'—C5'—C4'	120.2 (12)
C10—C5—C4	124.4 (6)	C10'—C5'—C4'	122.3 (10)
C7—C6—C5	121.9 (7)	C5'—C6'—C7'	120.2 (12)
C7—C6—H6	119.0	C5'—C6'—H6'	119.9
C5—C6—H6	119.0	C7'—C6'—H6'	119.9
C6—C7—C8	120.6 (6)	C8'—C7'—C6'	122.9 (9)
C6—C7—H7	119.7	C8'—C7'—H7'	118.6
C8—C7—H7	119.7	C6'—C7'—H7'	118.5
C9—C8—C7	120.7 (6)	C7'—C8'—C9'	120.3 (9)
C9—C8—H8	119.6	C7'—C8'—H8'	119.8
C7—C8—H8	119.6	C9'—C8'—H8'	119.9
C8—C9—C10	117.4 (7)	C10'—C9'—C8'	113.6 (10)
C8—C9—H9	121.3	C10'—C9'—H9'	123.2
C10—C9—H9	121.3	C8'—C9'—H9'	123.2
C1—C10—C5	113.4 (8)	C5'—C10'—C1'	119.3 (10)
C1—C10—C9	124.9 (9)	C5'—C10'—C9'	123.6 (10)
C5—C10—C9	121.6 (7)	C1'—C10'—C9'	116.6 (11)
N2—C11—N1	115.4 (9)	N2'—C11'—N1'	118.4 (15)
N2—C11—S1	125.6 (9)	N2'—C11'—S1'	120.8 (16)
N1—C11—S1	118.1 (10)	N1'—C11'—S1'	118.6 (18)
O1—C12—N1	122.6 (12)	O1'—C12'—N1'	121 (2)
O1—C12—C13	125.0 (14)	O1'—C12'—C13'	121 (2)
N1—C12—C13	110.6 (11)	N1'—C12'—C13'	115 (2)
C12—C13—C18	112.6 (11)	C14'—C13'—C18'	113 (2)
C12—C13—C14	110.4 (10)	C14'—C13'—C12'	108 (2)
C18—C13—C14	110.4 (11)	C18'—C13'—C12'	114 (2)
C12—C13—H13	107.7	C14'—C13'—H13'	107.2
C18—C13—H13	107.8	C18'—C13'—H13'	107.2
C14—C13—H13	107.7	C12'—C13'—H13'	107.2
C13—C14—C15	112.9 (10)	C13'—C14'—C15'	111 (2)
C13—C14—H14A	109.0	C13'—C14'—H14C	109.3
C15—C14—H14A	109.0	C15'—C14'—H14C	109.3
C13—C14—H14B	109.0	C13'—C14'—H14D	109.4
C15—C14—H14B	109.0	C15'—C14'—H14D	109.3
H14A—C14—H14B	107.8	H14C—C14'—H14D	108.0
C14—C15—C16	109.0 (8)	C16'—C15'—C14'	112.4 (17)
C14—C15—H15A	109.9	C16'—C15'—H15C	109.1
C16—C15—H15A	109.9	C14'—C15'—H15C	109.1
C14—C15—H15B	109.9	C16'—C15'—H15D	109.1
C16—C15—H15B	109.9	C14'—C15'—H15D	109.1
H15A—C15—H15B	108.3	H15C—C15'—H15D	107.9
C17—C16—C15	112.9 (11)	C17'—C16'—C15'	106 (2)
C17—C16—H16A	109.0	C17'—C16'—H16C	110.6
C15—C16—H16A	109.0	C15'—C16'—H16C	110.6
C17—C16—H16B	109.0	C17'—C16'—H16D	110.5
C15—C16—H16B	109.0	C15'—C16'—H16D	110.6
H16A—C16—H16B	107.8	H16C—C16'—H16D	108.7
C16—C17—C18	108.3 (9)	C16'—C17'—C18'	115.5 (19)
C16—C17—H17A	110.0	C16'—C17'—H17C	108.4
C18—C17—H17A	110.0	C18'—C17'—H17C	108.4
C16—C17—H17B	110.0	C16'—C17'—H17D	108.4
C18—C17—H17B	110.0	C18'—C17'—H17D	108.4
H17A—C17—H17B	108.4	H17C—C17'—H17D	107.5
C13—C18—C17	112.9 (9)	C13'—C18'—C17'	109 (2)
C13—C18—H18A	109.0	C13'—C18'—H18C	109.9
C17—C18—H18A	109.0	C17'—C18'—H18C	109.9
C13—C18—H18B	109.0	C13'—C18'—H18D	109.9
C17—C18—H18B	109.0	C17'—C18'—H18D	109.9
H18A—C18—H18B	107.8	H18C—C18'—H18D	108.3
C12—N1—C11	124.0 (12)	C11'—N2'—C1'	114.4 (14)
C12—N1—H1A	117 (3)	C11'—N2'—H2A	110 (3)
C11—N1—H1A	107 (3)	C1'—N2'—H2A	96 (3)
C11—N2—C1	120.8 (9)	C11'—N1'—C12'	117 (2)
C11—N2—H2A	113 (3)	C11'—N1'—H1A	106 (3)
C1—N2—H2A	119 (3)	C12'—N1'—H1A	108 (3)
			
C10—C1—C2—C3	−2 (3)	C10'—C1'—C2'—C3'	1 (4)
N2—C1—C2—C3	178.3 (12)	N2'—C1'—C2'—C3'	179 (3)
C1—C2—C3—C4	1.6 (19)	C1'—C2'—C3'—C4'	−1 (4)
C2—C3—C4—C5	−0.9 (17)	C2'—C3'—C4'—C5'	2 (3)
C3—C4—C5—C6	180.0 (11)	C3'—C4'—C5'—C6'	177 (3)
C3—C4—C5—C10	0 (2)	C3'—C4'—C5'—C10'	−3 (4)
C10—C5—C6—C7	0 (2)	C10'—C5'—C6'—C7'	0 (4)
C4—C5—C6—C7	180.0 (14)	C4'—C5'—C6'—C7'	180 (3)
C5—C6—C7—C8	0 (2)	C5'—C6'—C7'—C8'	−3 (5)
C6—C7—C8—C9	−3 (3)	C6'—C7'—C8'—C9'	11 (5)
C7—C8—C9—C10	6 (3)	C7'—C8'—C9'—C10'	−16 (4)
C2—C1—C10—C5	1 (3)	C6'—C5'—C10'—C1'	−177 (3)
N2—C1—C10—C5	−178.9 (14)	C4'—C5'—C10'—C1'	3 (4)
C2—C1—C10—C9	177.1 (18)	C6'—C5'—C10'—C9'	−5 (4)
N2—C1—C10—C9	−3 (3)	C4'—C5'—C10'—C9'	174 (3)
C6—C5—C10—C1	180.0 (16)	C2'—C1'—C10'—C5'	−2 (4)
C4—C5—C10—C1	0 (3)	N2'—C1'—C10'—C5'	180 (2)
C6—C5—C10—C9	4 (2)	C2'—C1'—C10'—C9'	−174 (3)
C4—C5—C10—C9	−176.5 (16)	N2'—C1'—C10'—C9'	7 (3)
C8—C9—C10—C1	178 (2)	C8'—C9'—C10'—C5'	13 (4)
C8—C9—C10—C5	−7 (3)	C8'—C9'—C10'—C1'	−175 (3)
O1—C12—C13—C18	24 (2)	O1'—C12'—C13'—C14'	−78 (3)
N1—C12—C13—C18	−170.9 (14)	N1'—C12'—C13'—C14'	123 (3)
O1—C12—C13—C14	−100.1 (15)	O1'—C12'—C13'—C18'	48 (4)
N1—C12—C13—C14	65.1 (18)	N1'—C12'—C13'—C18'	−111 (3)
C12—C13—C14—C15	179.2 (11)	C18'—C13'—C14'—C15'	53 (3)
C18—C13—C14—C15	54.0 (16)	C12'—C13'—C14'—C15'	179.9 (19)
C13—C14—C15—C16	−54.8 (15)	C13'—C14'—C15'—C16'	−57 (3)
C14—C15—C16—C17	57.3 (14)	C14'—C15'—C16'—C17'	57 (3)
C15—C16—C17—C18	−57.7 (13)	C15'—C16'—C17'—C18'	−59 (3)
C12—C13—C18—C17	−178.7 (11)	C14'—C13'—C18'—C17'	−51 (3)
C14—C13—C18—C17	−54.8 (16)	C12'—C13'—C18'—C17'	−175 (2)
C16—C17—C18—C13	56.1 (14)	C16'—C17'—C18'—C13'	56 (3)
O1—C12—N1—C11	−27 (2)	N1'—C11'—N2'—C1'	−120 (2)
C13—C12—N1—C11	167.2 (13)	S1'—C11'—N2'—C1'	43 (2)
N2—C11—N1—C12	23 (2)	C2'—C1'—N2'—C11'	66 (4)
S1—C11—N1—C12	−167.5 (12)	C10'—C1'—N2'—C11'	−116.0 (19)
N1—C11—N2—C1	159.0 (11)	N2'—C11'—N1'—C12'	−43 (3)
S1—C11—N2—C1	−9.9 (13)	S1'—C11'—N1'—C12'	153.6 (19)
C10—C1—N2—C11	−80 (2)	O1'—C12'—N1'—C11'	46 (4)
C2—C1—N2—C11	99.7 (12)	C13'—C12'—N1'—C11'	−156 (2)

### Hydrogen-bond geometry (Å, º)

Cg1 is the centroid of the C1–C5/C10 ring.

**Table:**

*D*—H···*A*	*D*—H	H···*A*	*D*···*A*	*D*—H···*A*
N2—H2*A*···O1	0.88 (4)	1.97 (4)	2.667 (15)	135 (4)
N2′—H2*A*···O1′	0.93 (4)	2.03 (4)	2.62 (3)	120 (4)
N1—H1*A*···S1^i^	0.87 (3)	2.53 (3)	3.370 (19)	161 (4)
N1′—H1*A*···S1′^i^	0.90 (4)	2.59 (4)	3.44 (3)	159 (4)
C18—H18*B*···*Cg*1^ii^	0.90	2.66	3.527 (2)	148

Symmetry codes: (i) −*x*+2, −*y*, −*z*+1; (ii) −*x*+2, −*y*+1, −*z*+1.
