# Supplementary figures and images for: Crystal structure of N-[(naphthalen-1-yl)carbamo­thio­yl]cyclo­hexa­necarboxamide
Source: Acta Crystallogr E Crystallogr Commun. 2015 Jun 27;71(Pt 7):o508–9. doi: 10.1107/S2056989015011950 (PMC4518972; doi:10.1107/S2056989015011950)

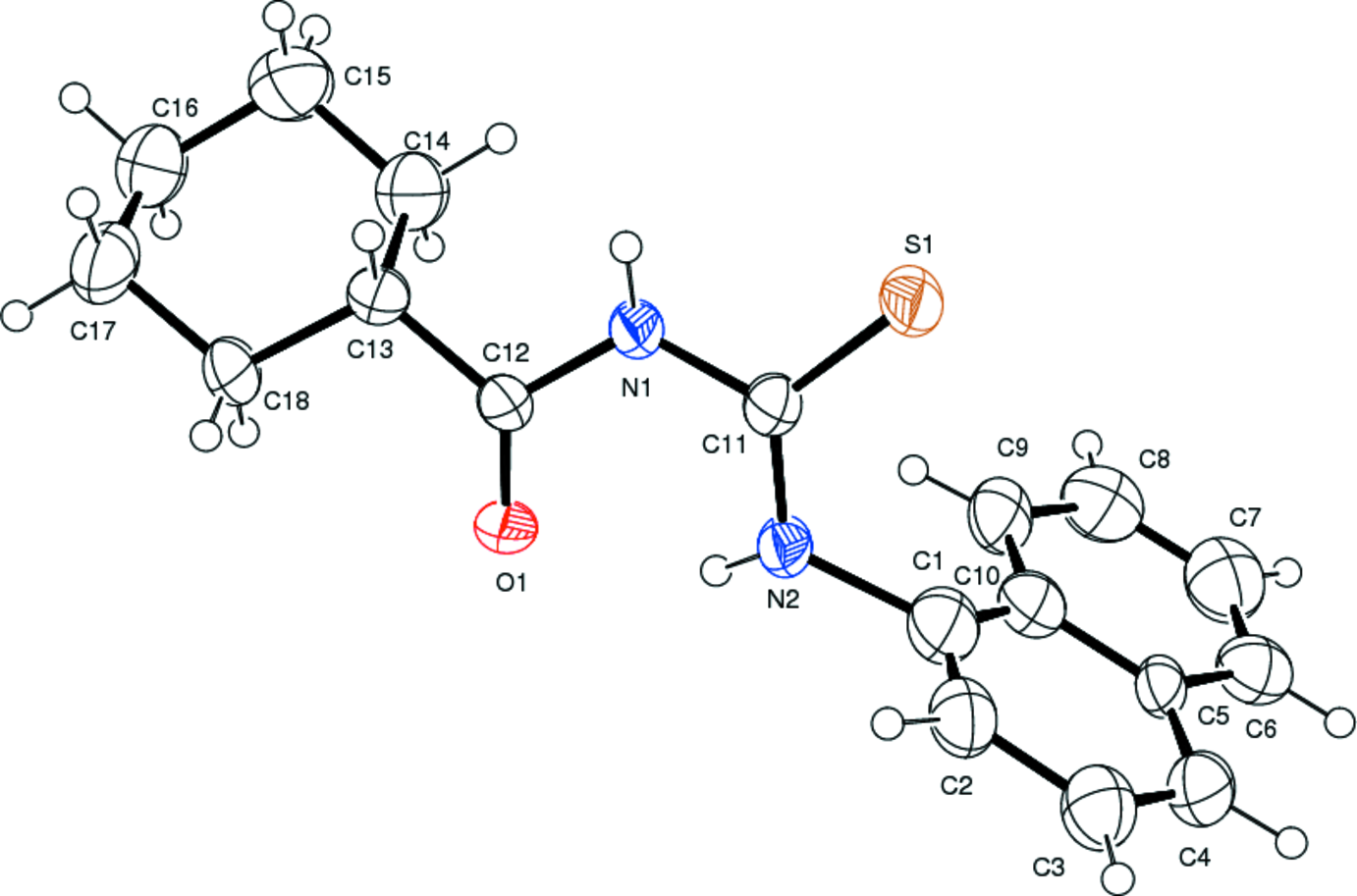

Supplement: Supplementary file 4 [file e-71-0o508-fig1.tif]

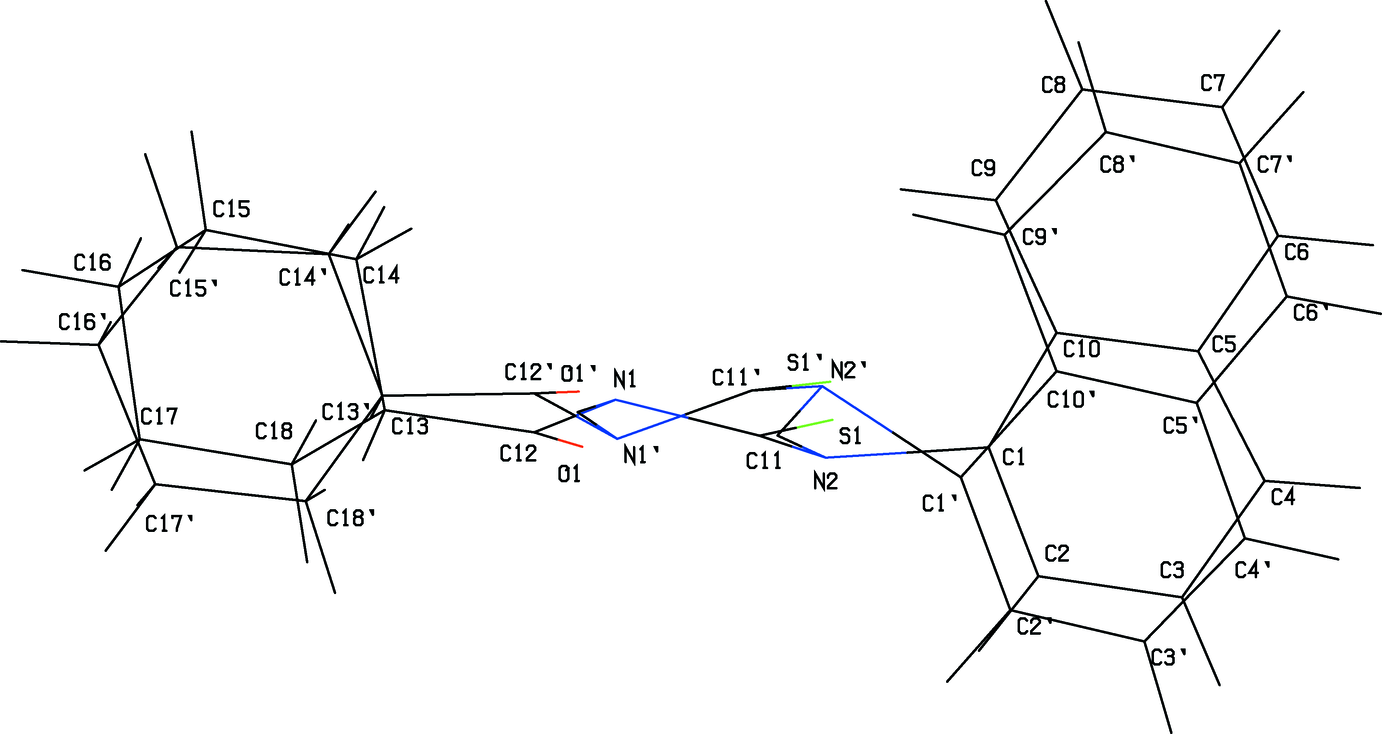

Supplement: Supplementary file 5 [file e-71-0o508-fig2.tif]

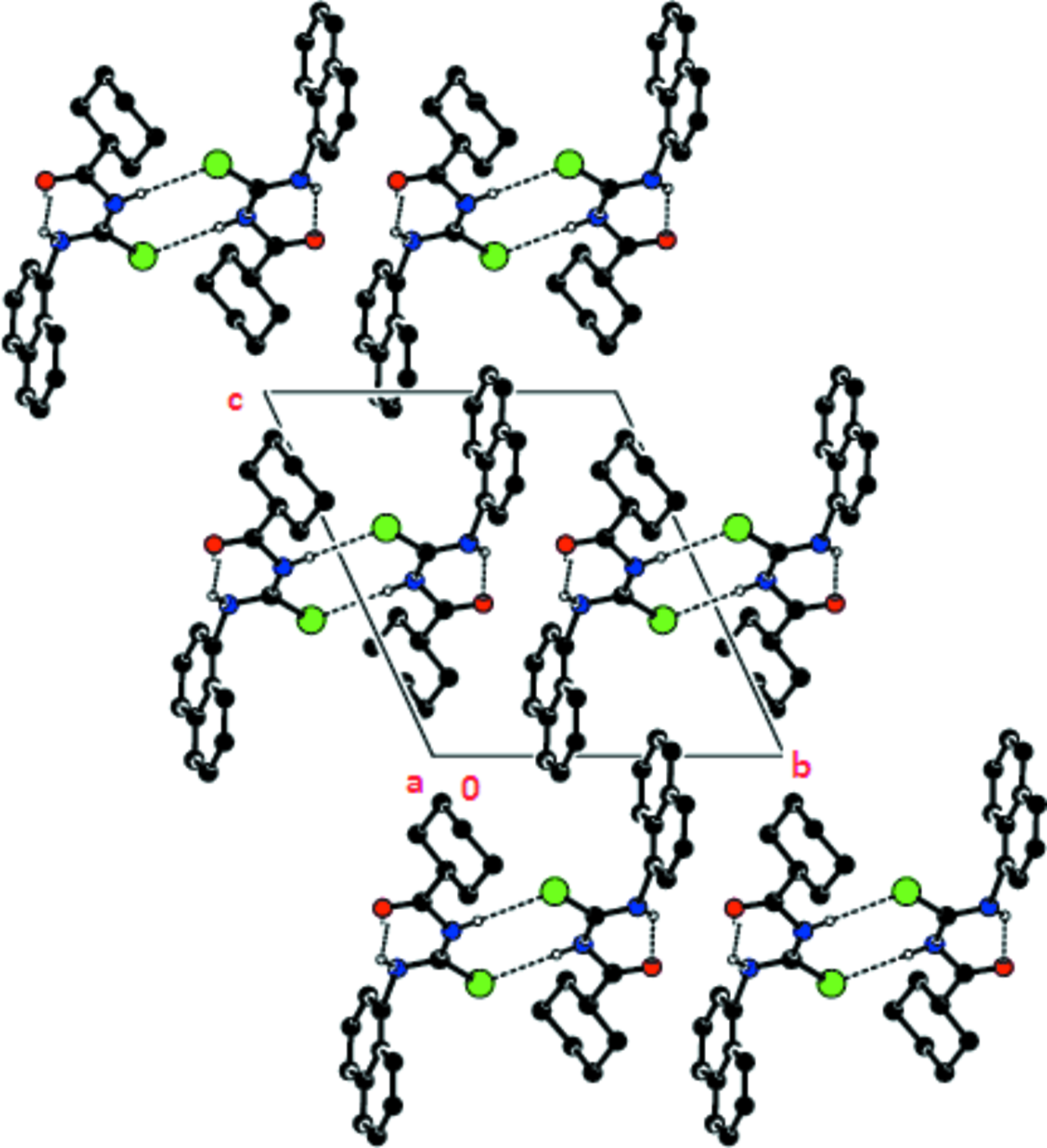

Supplement: Supplementary file 6 [file e-71-0o508-fig3.tif]
